# Anesthetic Management for Laser Excision of Ball-Valving Laryngeal Masses

**DOI:** 10.1155/2015/875053

**Published:** 2015-05-24

**Authors:** Benjamin B. Bruins, Natasha Mirza, Ernest Gomez, Joshua H. Atkins

**Affiliations:** ^1^Department of Anesthesiology & Critical Care, Hospital of the University of Pennsylvania, Philadelphia, PA 19104, USA; ^2^Department of Otorhinolaryngology, Head and Neck Surgery, Perelman School of Medicine, The University of Pennsylvania, Philadelphia, PA 19104, USA; ^3^Department of Otorhinolaryngology, Head and Neck Surgery, Hospital of the University of Pennsylvania, Philadelphia, PA 19104, USA; ^4^Department of Anesthesiology & Critical Care, Department of Otorhinolaryngology, Head and Neck Surgery, Perelman School of Medicine, The University of Pennsylvania, PA 19104, USA

## Abstract

A 47-year-old obese woman with GERD and COPD presents for CO_2_-laser excision of bilateral vocal fold masses. She had a history of progressive hoarseness and difficulty in breathing. Nasopharyngeal laryngoscopy revealed large, mobile, bilateral vocal cord polyps that demonstrated dynamic occlusion of the glottis. We describe the airway and anesthetic management of this patient with a topicalized C-MAC video laryngoscopic intubation using a 4.5 mm Xomed Laser Shield II endotracheal tube. We examine the challenges of anesthetic management unique to the combined circumstances of a ball-valve lesion and the need for a narrow-bore laser compatible endotracheal tube.

## 1. Introduction

Airway management of the patient with a glottic lesion producing dynamic total airway occlusion (ball-valve effect) requires a specialized management plan [1]. Induction of general anesthesia, loss of hypopharyngeal tone, abolition of spontaneous ventilation, and initiation of positive pressure ventilation can result in the inability to ventilate and/or intubate. For these reasons, airway management in patients with large or periglottic airway masses is often accomplished with awake or minimally sedated fiberoptic bronchoscopic endotracheal tube placement [2, 3]. The added requirement for laser surgery at the glottis around a narrow-bore (<6 mm) endotracheal tube limits this approach. We describe successful topicalized-sedated intubation with Storz C-MAC (Karl Storz, Tuttlingen, Germany) videolaryngoscopy and characterize technical limitations associated with blind techniques and laser tubes.

The patient gave written consent for publication of the details of the case.

## 2. Case Description

A 47-year-old, obese (BMI 33) woman with GERD, COPD, and 60-pack year smoking history presented to the operative theater for CO_2_-laser excision of bilateral vocal fold polyps. Home medications included a proton pump inhibitor and inhaled beta-2 agonist. Preoperative nasopharyngeal laryngoscopy demonstrated a left highly mobile polyp that herniated from a supraglottic position during expiration to a subglottic position during inspiration and a smaller right polyp. Airway occlusion was estimated at 70–80% by the surgical team as depicted in video 1 in Supplementary Material available online at http://dx.doi.org/10.1155/2015/875053. The patient declined tracheostomy and was scheduled for an elective resection of the polyps in the operating room. Physical examination revealed full neck mobility, the ability to sublux her mandible, intact dentition, 3 cm mentohyoid distance, 4 cm interincisor distance, and Mallampati score of 1. History was significant for hoarseness and an exaggerated gag reflex. The surgical plan was mass excision employing the CO_2_-laser and a 4.5 mm laser-resistant endotracheal tube was requested to facilitate exposure.

Nebulized lidocaine (2%, 8 mL, 8 L/min) was administered in the holding area over 15 minutes. The patient was brought to the operating room, positioned 40 degrees head-up on the operating room table, ASA standard monitors were applied, and ondansetron (4 mg) was administered. Nasal cannula oxygen (4 L/min) with end-tidal CO_2_ sampling was placed. A bolus of intravenous dexmedetomidine (1 mcg/kg) was administered over 10 minutes during which a remifentanil infusion was started (0.05 mcg/kg/min). An Ovassapian intubating oral airway with a dollop of 5% lidocaine ointment on the tip was placed and allowed to sit for 5 minutes. A first attempt at laryngoscopy with a C-MAC D-Blade produced gagging, so additional lidocaine 2% (4 mL) was administered to the posterior oropharynx with a MADgic atomizer (Teleflex Medical, Research Triangle Park, NC) and a 0.2 mcg/kg bolus of remifentanil delivered. The CMAC D-Blade was reintroduced with minimal coughing to reveal a grade I view of the glottis and the lesions. A 4.5 mm ID Xomed Laser Shield II endotracheal tube (Medtronic Xomed, Jacksonville, FL) with stylet was passed through the cords under indirect visualization. The endotracheal tube cuff was inflated to a minimal occlusion volume with methylene blue tinted normal saline. General anesthesia was induced with propofol and remifentanil and maintained with those agents and a background dexmedetomidine infusion (0.3 mcg/kg/h). Empiric dexamethasone (10 mg) was administered to limit airway edema. Additional measures to minimize risk of airway fire included reduction of FiO_2_ to 40% and the use of moist gauze in and around the airway.

Suspension laryngoscopy and CO_2_-laser excision commenced. During the repositioning of the surgical laryngoscope for resection of the second lesion the patient developed two episodes of profound bradycardia that evolved to transient asystole. This was effectively treated with immediate removal of the laryngoscope, sixty seconds of CPR, intravenous atropine (0.5 mg), and cessation of dexmedetomidine infusion. Suspension laryngoscopy was repeated and the second lesion was resected without further event. The patient emerged smoothly from anesthesia, evidenced no postoperative respiratory distress, and required no additional medications (e.g., racemic epinephrine and albuterol) for airway maintenance. The patient was wide-awake 30 minutes after surgery and expressed a strong preference for the same-day discharge and signed out against medical advice.

## 3. Discussion

Airway management of the patient with a glottic lesion producing dynamic, airway occlusion (ball-valve effect) requires a specialized management plan [1]. Induction of general anesthesia, loss of hypopharyngeal tone, abolition of spontaneous ventilation, and initiation of positive pressure ventilation can promote total airway obstruction and result in the inability to ventilate and/or intubate. Traditional airway rescue techniques of cricothyrotomy or LMA will be limited in the event of an obstructing into the glottis. Awake tracheostomy may be considered in this setting, but the patient refused this option and the surgeon was confident that a near total resection was feasible without the associated morbidity of a tracheostomy. While we have previously described the technique of subglottic HFJV [4], in light of the complexity of the resection, the anticipated duration, and the pulmonary comorbidities of the patient, the team elected endotracheal intubation.

Airway management of the “difficult airway” is often accomplished with awake or minimally sedated fiberoptic bronchoscopic intubation. The added requirement for laser surgery at the glottis around a narrow-bore (<6 mm) endotracheal tube with a mobile glottic lesion limits the appeal of this approach. The “blind” translation of an endotracheal tube over a gum-elastic bougie, tube exchange catheter (e.g., if downsizing to a smaller tube), or fiberoptic scope is commonly associated with tube impingement on the arytenoid or vocal folds [5]. Such an approach also risks obstruction or bleeding into the airway should the tube encounter the airway lesions during passage.

**Figure 1:**
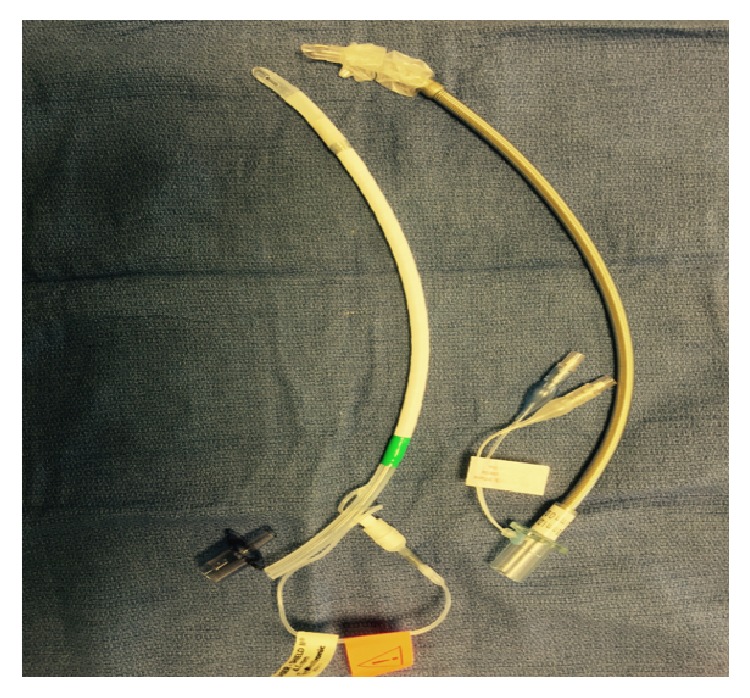
4.5 mm ID Xomed Laser Shield II (left) and laser-safe (right) endotracheal tubes.

Selection of the appropriate laser-resistant endotracheal tube is of utmost importance. The Xomed Laser Shield Tube II (Medtronic, Minneapolis, MN) is made of silicone rubber wrapped with aluminum and Teflon (see Figure 1) and offers several advantages. In contrast with the Laser Flex endotracheal tube (Mallinckrodt, St. Louis, MO), the connector is removable and the pilot balloon connectors are external to the lumen allowing a 2.9 mm fiberoptic bronchoscope to tightly translate through even the smallest 4.5 mm ID tube. Additionally, the Xomed Laser Shield Tube II is more pliable which facilitates manipulation and may limit tube related trauma to friable lesions.

Sustained airway obstruction is unlikely in the spontaneously ventilating patient under minimal sedation. Surgical direct laryngoscopy (MDL) with introduction of a cannula across the lesion for oxygen delivery would be one rescue option. Rigid bronchoscopy and formal awake tracheostomy should be considered as alternative strategies. Awake videolaryngoscopy (VL) offers a safe and practical approach to intubation, preserving the ability to visualize the glottis, the lesion, and movements of the endotracheal tube during intubation. The entire surgical and anesthesia team can follow the intubation in an effort to maximize safety and facilitate immediate expert input should intubation prove challenging. A growing body of evidence supports awake VL or combined awake VL and fiberoptic guidance for complex airway management [6–9]. Each respective VL technique offers unique advantages. For a glottic lesion it is important to visualize the tip of the blade as it transits towards the larynx. The GlideScope (Verathon Medical, Bothell, WA) does not allow for continuous distal tip visualization [10]. Stylet options are also important. The traditional rigid GlideScope stylet, recommended for most intubations, cannot pass through a 4.5 ID laser tube. Some devices such as the PENTAX Airway Scope (AWS, Ambu A/S, Ballerup, Denmark) offer an intubating channel that eliminates the need for a stylet altogether but are recommended to use with a straight blade approach that might not be suitable for all lesions. Similarly when using some devices it can be challenging to reliably introduce suction directly to the area of the glottis to clear bleeding or secretions during laryngoscopy. We selected the Storz CMAC VL for the ability to continuously visualize the tip of the blade, reliably deliver surgical suction to the glottis if bleeding should occur, and use a narrow, malleable stylet if needed.

Various approaches to patient sedation and airway preparation for awake laryngoscopy have been described and largely parallel those adopted for fiberoptic intubation. Dexmedetomidine is a useful sedative in this setting as respiratory depression is minimal and patients retain the ability to follow commands, including deep inspiration and expiration that promote opening of the airway. Dexmedetomidine decreases sympathetic circulating norepinephrine levels and is associated with bradycardia [11]. Remifentanil is also associated with bradycardia and laryngoscopy exerts a profound stimulus of vagal activity. We believe that the drug combination along with the self-reported history of a profound gag reflex and the receding effects of topical lidocaine 60 minutes into the procedure contributed to the asystole. Pretreatment with an anticholinergic agent should be considered when the combination of dexmedetomidine and remifentanil is used for suspension laryngoscopy.

## Supplementary Material

Supplementary material. Video shows the nasopharyngolaryngoscopy (NPL) exam performed in the clinic by the ENT surgeon without sedation. The video demonstrates the ball-valving effect of the lesions with significant, intermittent obstruction of the glottis.
